# Provider experiences with improvised uterine balloon tamponade for the management of uncontrolled postpartum hemorrhage in Kenya

**DOI:** 10.1016/j.ijgo.2016.05.006

**Published:** 2016-11

**Authors:** Abirami Natarajan, Anna Alaska Pendleton, Brett D. Nelson, Roy Ahn, Monica Oguttu, Lidu Dulo, Melody J. Eckardt, Thomas F. Burke

**Affiliations:** a Division of Global Health and Human Rights, Department of Emergency Medicine, Massachusetts General Hospital, Boston, MA, USA; b Harvard Medical School, Boston, MA, USA; c Kisumu Medical Education Trust, Kisumu, Kenya

**Keywords:** Kenya, Maternal health, Maternal hemorrhage, Maternal mortality, Postpartum hemorrhage, Uterine balloon tamponade

## Abstract

**Objective:**

To understand healthcare providers’ experiences with improvised uterine balloon tamponade (UBT) for the management of uncontrolled postpartum hemorrhage (PPH).

**Methods:**

In a qualitative descriptive study, in-depth semi-structured interviews were conducted between November 2014 and June 2015 among Kenyan healthcare providers who had previous experience with improvising a UBT device. Interviews were conducted, audio-recorded, and transcribed.

**Results:**

Overall, 29 healthcare providers (14 nurse-midwifes, 7 medical officers, 7 obstetricians, and 1 clinical officer) were interviewed. Providers perceived improvised UBT as valuable for managing uncontrolled PPH. Reported benefits included effectiveness in arresting hemorrhage and averting hysterectomy, and ease of use by providers of all levels of training. Providers used various materials to construct an improvised UBT. Challenges to improvising UBT—e.g. searching for materials during an emergency, procuring male condoms, and inserting fluid via a small syringe—were reported to lead to delays in care. Providers described their introduction to improvised UBT through both formal and informal sources. There was universal enthusiasm for widespread standardized training.

**Conclusion:**

Improvised UBT seems to be a valuable second-line treatment for uncontrolled PPH that can be used by providers of all levels. UBT might be optimized by integrating a standard package across the health system.

## 1 Introduction

Uncontrolled postpartum hemorrhage (PPH) is the leading cause of maternal mortality worldwide and accounts for 33% of maternal deaths in Sub-Saharan Africa [1], [2]. Recently, there has been increasing support for the use of uterine balloon tamponade (UBT) as a second-line treatment for uncontrolled PPH when medical management fails [3], [4], [5], [6].

A recent multicountry case series conducted in Kenya, Sierra Leone, Senegal, and Nepal [7] demonstrated that the PPH package called “Every Second Matters for Mothers and Babies” (ESM-UBT)—which includes a 3-hour training session, checklists, wall charts, and prepackaged UBT kits—effectively arrested PPH at every level of the health system. A standard prepackaged UBT kit comprises the materials required to assemble a UBT, including a condom, urinary catheter, string, and 50-mL syringe. An emergent finding from this case series was that informal awareness of, and skills development for, UBT use spread rapidly beyond the intended implementation of the ESM-UBT package. Consequently, maternal health providers periodically made improvised UBT devices using materials locally available at their own facilities.

In the multicountry case series [7], women with uncontrolled PPH survived 98% of the time if they presented at facilities that were “on-line” with ESM-UBT, where on-line was defined as providers that were fully trained with checklists and wall charts in place, and ready access to prepackaged ESM-UBT kits. However, the survival of women with uncontrolled PPH decreased to 83% for those attending a facility where an improvised UBT device was used [7].

Current research on UBT has focused predominantly on quantitative outcomes related to the effectiveness of UBT use, including bleeding cessation and patient survival [7]. The apparent differences in outcome between women with uncontrolled PPH who received care at facilities on-line with ESM-UBT and those who received an improvised UBT device raise important questions regarding optimal provider-, patient-, and training-related factors. Although a few studies have examined provider perspectives on the use of prepackaged UBT kits after comprehensive training [8], no studies have examined the experiences of providers who have resorted to improvised UBT devices as far as we are aware. The aim of the present study was therefore to understand provider perspectives and experiences with improvising a UBT device at health facilities in Kenya.

## 2 Materials and methods

In a qualitative study, in-depth interviews were conducted between November 1, 2014, and June 30, 2015, to understand the experiences of healthcare providers who used an improvised UBT device for the management of uncontrolled PPH in Kenya. Ethical approval was obtained from the Partners Healthcare Human Research Committee (Boston, MA, USA), the Maseno University Ethics Review Committee (Maseno, Kenya), and the Ministry of Health of Kenya. All providers gave verbal informed consent.

The healthcare providers were selected using snowball sampling. Purposive sampling was undertaken to ensure that different levels of healthcare providers from different regions were represented. Healthcare providers were interviewed until thematic saturation was achieved.

Semi-structured interviews (lasting 15–70 minutes) at the providers’ healthcare facilities explored experiences and perspectives of improvised UBT use. Interviews were conducted in English (all interviewed providers were native English speakers), audio recorded, and transcribed after the interview. Providers who were in regions that were deemed unsafe for researchers were interviewed by telephone, as were those who were unavailable at their facility during the research visit. Healthcare providers were assessed on their training on UBT, experiences with improvising and using an improvised UBT device, challenges, and recommendations for improvement (Box 1).

Data were analyzed using a validated iterative thematic coding method [9]. Two researchers (A.N. and A.A.P.) independently read the transcripts and developed the code. A codebook was agreed, and interviews were subsequently recoded using NVivo 10 (QSR International, Cambridge, MA, USA). Provider comments were organized into three broad domains: perception and experiences of improvised UBT, challenges to improvisation, and opportunities for improvement.

## 3 Results

During the study period, 29 providers who had improvised a UBT device for uncontrolled PPH were identified and interviewed. The interviewed providers included 14 nurse-midwives, seven obstetricians, seven medical officers (doctors who had completed 1 year of internship), and one clinical officer. Table 1 describes both the work experience of each type of healthcare provider, and their reported experience with improvising an UBT device from local materials. Representative quotations from the interviews are included in Supplementary Material S1.

Regarding the interviewed providers’ experiences with improvising an UBT device, the most commonly described benefits included effectiveness in arresting hemorrhage (reported by 27 of the 29 providers), averting hysterectomy (8/29), minimal expertise needed to use the device (6/29), and widespread availability of materials (4/29). All the interviewed providers regarded improvised UBT as a valuable and useful addition to other techniques for PPH management. All the providers described using improvised UBT as a second-line treatment for uncontrolled PPH only after they had excluded treatable causes of PPH and had administered multiple doses of uterotonics. Providers reported using the improvised UBT device in various situations, including as a primary endpoint, before referral, and as an alternative to hysterectomy. Furthermore, providers reported inserting an improvised UBT device in a wide range of clinical cases, from women who were clinically stable to women who showed signs and symptoms of severe hemorrhagic shock. Two of the 29 providers explicitly described the utility of improvised UBT as life-saving when women were critically ill with no further options available.

Thirteen of the interviewed providers were based in hospitals with surgical capabilities, all of whom described improvised UBT as an important alternative to hysterectomy. Use of an improvised UBT device instead of performing an emergency hysterectomy allowed preservation of a woman’s uterus, reduced the risks inherent in emergency surgery, and facilitated more timely management of uncontrolled PPH. Two providers described successfully inserting an improvised UBT device in situations when a surgeon was unavailable or when a patient was not a surgical candidate. Improvised UBT was widely accepted among the seven obstetricians as a less invasive method of managing PPH. Six of the seven obstetricians reported that they either perceived UBT as an alternative to hysterectomy or performed emergency hysterectomies for uncontrolled PPH considerably less frequently subsequent to integrating improvised UBT into their practice.

Overall, providers improvised a UBT device using a range of materials and techniques. Providers most commonly reported using the condom–catheter improvised UBT method to treat uncontrolled PPH. Two providers reported using a Foley catheter (sometimes multiple) as an improvised UBT device and inflating the catheter with 50–70 mL of water. When assembling a condom–catheter improvised UBT, providers used materials available at their facilities including male condom, catheter (most commonly an 18- or 20-mL Foley catheter), string, saline, and water. One provider used a surgical glove instead of a condom. The method of fluid infusion varied and ranged from gravity inflation using an intravenous tubing set to a syringe (providers most commonly used a 10-mL or 20-mL syringe to inflate the balloon). Once the improvised UBT was inside the uterus, providers reported filling the balloon with fluid, ranging from 250 mL to 2000 mL. Providers reported using suture, string, cap of a needle, forceps clamps, and 5-mL syringes to prevent fluid from leaking from the inflated device.

Several challenges to using the improvised UBT during the time of hemorrhage emerged. Providers consistently described their experience of managing uncontrolled PPH as a chaotic and stressful event. Although 10 of the 29 providers reported taking measures to carefully create PPH emergency trays containing all the materials required to improvise a UBT device, 19 providers were unprepared for assembly of an improvised UBT device and faced the challenge of obtaining materials in the midst of an emergent situation, often while the patient was actively hemorrhaging. Nearly one-third of providers described rushing around to different departments of their facility to gather materials to make up an improvised UBT device. This was most challenging when providers were alone during the night or when materials were locked away, rendering them inaccessible. In some cases, delays due to UBT device improvisation were prolonged by up to 20 minutes. Three providers additionally described the challenge of obtaining male condoms, which were not available in the maternity ward. Maternal health providers reported using either an intravenous bag of solution and infusion set to fill the improvised uterine balloon via gravity or a syringe; however, six of the 29 providers had access to only small syringes (10–20 mL) and described the considerable challenge of using a small volume to inflate an improvised UBT device with fluid.

Of the 29 providers, two described experiences with women who died after improvised UBT insertion. In both situations, there had been significant delays in transporting the hemorrhaging woman before placement of the improvised UBT device, and the devices were inserted at the referral facility only after the woman was moribund and had been in advanced shock for a prolonged period of time. In both cases, providers reported use of the standard protocol for managing PPH (i.e. multiple doses of uterotonics and intravenous fluids) before using improvised UBT. Providers at the receiving facilities felt that improved awareness of improvised UBT devices at the referring facility and earlier insertion of the improvised balloon before referral might have saved the lives of these two women.

Providers reported that awareness of the use of an improvised UBT device for the management of uncontrolled PPH was increasing. Four of the seven obstetricians reported theoretic instruction of UBT during in-service training, but no one reported practical experience, or had integrated UBT into their clinical practice, before August 2012 (i.e. when ESM-UBT was implemented in Kenya). Approximately half the providers (14/29) had attended formal training on UBT; however, the described curriculum varied with respect to the length of didactic teaching versus hands-on practical learning. The remaining 15 providers had improvised a UBT device after either informal discussions with colleagues or observation of other providers utilizing an improvised UBT device.

Another theme that emerged was the desire for widespread, standardized, formal training on use of UBT, particularly in lower-level facilities. Providers consistently reported that hands-on practice (especially during real-life situations) was critical for their confidence in assembling an improvised UBT device. Four of the 29 providers reported that they understood the general principles behind UBT, but were uncertain about the exact indications for placement, the most efficient assembly, and when the UBT device should be removed*.*

Six providers had prior experience with both improvising a UBT device and using a prepackaged ESM-UBT kit. These providers perceived the ESM-UBT kit to be more efficient than an improvised UBT device, easier to use, and faster. Furthermore, providers who had pre-gathered improvised UBT materials into an emergency PPH tray reported preparedness in emergent situations. Additional provider recommendations included an emphasis on incorporating UBT into preservice training across all cadres of healthcare provider and especially for lower-level providers.

## 4 Discussion

With varying amounts of exposure, providers of all levels of training seemed able to improvise their own UBT devices and to incorporate them into their management pathway for uncontrolled PPH. All 29 of the interviewed providers perceived the improvised UBT device as valuable, minimally invasive, and effective for managing uncontrolled PPH. Although the interviewed providers were resourceful in their ability to improvise a UBT device using a wide variety of locally available materials, delays in assembly at the time of critical need were universally reported. Lastly, there was significant desire for increasing access to quality training including hands-on skills development.

Previous studies have provided insights into provider experiences of using UBT after the provision of focused training, educational materials, and prepackaged UBT kits [7]. To our knowledge, however, no studies have examined the process, experiences, or challenges of impromptu improvisation of a UBT device without formal training or prepared materials. The present study identified specific factors including pre-emergency preparedness, practical experience, and useful materials currently unavailable at facilities (e.g. 60-mL syringe) that might lead to more efficient, timely, and optimal use of UBT in emergent situations.

The two maternal deaths described in the present study highlight the stark reality of what occurs when uncontrolled hemorrhage is not addressed in a timely fashion. Expanding standardized UBT training and access to prepared UBT kits—particularly at remote health facilities that might not have access to blood or surgical capabilities—represents an opportunity to potentially reduce morbidity and mortality among women with severe PPH.

In July 2013, UBT was formally added to the Kenyan national protocol for treatment of uncontrolled PPH. Integrating UBT into clinical practice has been slowly occurring via incorporation into emergency obstetric and newborn care training, focused UBT training sessions, addition to university curricula, and word of mouth among healthcare providers [10]. So far, however, few providers have undergone formal training and prepackaged UBT kits have not been widely distributed. As described previously, the survival of women with uncontrolled PPH decreased from 98% at facilities where healthcare providers were formally trained in UBT and prepackaged kits were available to 83% at facilities where an improvised UBT device was used [7]. Although the present study provides insight into the challenges of improvising a UBT device that might contribute to those preliminary findings, more rigorous studies regarding the effectiveness of improvised UBT, as compared with UBT or even more expensive preassembled devices such as the Bakri Balloon, are needed.

Given these findings, a standardized package should be scaled across all levels of the health system in Kenya and beyond. When preassembled kits are unavailable, providers should be encouraged to take the initiative, as some already have, to create PPH emergency trays with improvised UBTs that are readily accessible at the moment that uncontrolled PPH is recognized.

The present study has a few limitations. Interviews were based on provider recollection, which might be affected by social desirability or recall bias. This was partly mitigated by emphasizing that the interviews were designed only to improve the program and would not be used in any kind of review. Additionally, providers who have improvised a UBT device probably represent early adopters, and therefore are unlikely to be representative of all healthcare providers providing obstetric care.

In conclusion, improvised UBT is a valuable second-line treatment for uncontrolled PPH. Outcomes of uncontrolled PPH are likely to be optimized by means of uniform integration of a standard UBT package across health systems.

The following are the supplementary data related to this article.

Supplementary Material S1 Representative quotations of select themes. Supplementary Material S1

## Figures and Tables

**Table 1:** Characteristics of healthcare providers who improvised UBT kits.

Type of provider	No. interviewed	Mean length of work experience, y	Mean no. of times improvised UBT
Nurse-midwife	14	11.3	1.5
Clinical officer	1	6.0	3.0
Medical officer	7	5.0	3.0
Obstetrician	7	5.8	2.6

Abbreviation: UBT, uterine balloon tamponade.

## Box 1 Data collected and sample questions from the semi-structured interview

**General information**

Facility ownership, level of facility, deliveries per month.

Provider level of training, years of experience, total experience improvising uterine balloon tamponade (UBT).

**Provider perception**

Please describe your experience with improvising a UBT device.

What were the strengths of improvising a UBT device?

What are the challenges of improvising a UBT device?

How would preassembled UBT kits affect patient care?

How well did you think the improvised UBT device worked?

If you had to improvise again, how would you do it differently?

Were there any concerns you had with the improvised UBT device?

**Training**

When did you first learn about the UBT device?

Describe the type of training that you had.

Did you feel prepared to use the UBT device after your training?

Did you learn about the UBT device during preservice training?

How long was your training?

Was there a practical component?

**Process of improvisation**

Please walk us through the actual placement of the improvised UBT device.

What materials did you use?

How did the process of improvising go?

Were the materials readily available? Where did you get them?

How long did it take to fill the balloon? How much water did you use?

**Effect of balloon**

What was the effect of the improvised UBT device on the bleeding?

How did the clinical status of the mother change after UBT device placement?

Was the improvised UBT device ever displaced?

What other measures did you take (e.g., fluids, blood, antibiotics)?

Did you refer the mother? If so, why? If not, why?

**Improving UBT**

How could the experience of improvising a UBT device be improved?

What recommendations do you have?

Alt-text: Box 1
