# Supplementary material for: Provider experiences with improvised uterine balloon tamponade for the management of uncontrolled postpartum hemorrhage in Kenya
Source: Int J Gynaecol Obstet. 2016 Nov;135(2):210–3. doi: 10.1016/j.ijgo.2016.05.006 (PMC5073071; doi:10.1016/j.ijgo.2016.05.006)
Supplement: Supplementary Material S1 — Representative quotations of select themes. [file mmc1.docx]

**Supplemental Material**

**Providers described value of IUBT as a last resort to uncontrolled PPH in a wide range of situations**

*“It is lifesaving. The case that we used the balloon [IUBT device], in that situation, we had nothing else left to do. Every time we have used it since then, it has worked perfectly well. It became a routine…To me it was lifesaving procedure which we have adopted.”* – Obstetrician

*“In that situation, I don’t think that there is anything else that would work. I don’t know what I would have done if the improvised balloon tamponade had not worked. It is a very useful thing that everyone needs to know. I think that one advantage is that it will markedly reduce hemorrhage, because I am not sure what else I could have done, if it had failed.”* – Medical officer

*“At least at higher-level facilities there are obstetricians and surgical capabilities. So it could be more useful at lower-level facilities … Here we have the personnel, people can improvise. But if they are seen in lower-level facilities and they are referred here, that referral process is lost time. The time needed to save a mother. So you need to train people in even lower-level facilities.”* – Obstetrician

*“The advantages are that it {IUBT} reduces the chances of a patient going for hysterectomy. And it really, really improves the chances of managing PPH. Because sometimes we don’t have an anesthetist on call or we don’t have any blood. So instead of waiting for the anesthetist to come, we just improvise, and the bleeding just stops. It really, really affects the bleeding.”* – Obstetrician

*“It is something simple, and you can use it to save somebody’s life. When you don’t have blood, you just put the improvised UBT in, and it stops the bleeding. Because before we didn’t know what the issue was so people would go for hysterectomy, but it is something that is quite simple and it’s cheap, even if you are doing it using a condom. And it really* helps.” – Nurse-midwife

***Challenges to improvising UBT can lead to delay in care***

*“It became stressful, because it was my first time. I had not assembled the equipment before. So I had to run to get some of the equipment, and it was late in the evening. I was managing alone at that time. It took me several minutes. I think it was like 20 [minutes]. It was unfortunate that the mother was still bleeding at that time.”* – Nurse-midwife

*“We had not anticipated the event. We couldn’t find a string, we didn’t know where a condom was, we had to look for it at the maternal health clinic, so we had to call the nurse on call to come open the door for us, so it was a bit challenging. Very tricky assembling it unless you have all of those things prepared.”* – Obstetrician

*“It was cumbersome because I was assembling while the mother was bleeding. That was a harsh experience. You have to assemble the equipment, and the male condom may be very far from maternity. Maternity is not a place for condoms, you see. So I had to get it from the health center and then you go for the Foley catheter, and it is in the emergency tray. So you have to assemble this, the mother is pouring [blood], and you are alone in the facility. It was after work, actually it was in the evening, around 5:30 PM. Yes, and I can say it was cumbersome because the items were far apart. By the time you come, you have lost a sense of time, and that is how it became cumbersome. But if you assemble it earlier it is doable.”* – Nurse-midwife

*“From my experience, it was a bit hard, because at a mission hospital there is the issue with condoms. Accessing condoms is not easy within the labor ward. So you had to run up and down looking for a condom. The condom was only available at the comprehensive care center. So someone had to go up and bring that… Getting the condom was the biggest hassle; they were locked up and not easy to access. And they would ask you why do you need a condom.”* – Medical officer

*“It is so hard, because every time you are withdrawing. You can imagine how many withdraws I made for 1500 ml. Divide by 20 – you can withdraw for a long time. But with a bigger syringe, it will be easier.”* – Nurse-midwife

*“You know it is an improvisation and the package is not there. Not many health workers will think about finding a condom and catheter. You know in terms of improvising, you are using a 10 cc syringe, and that patient is bleeding. It would be much easier if you could come up with a bigger pump. Just imagine inserting one liter using a 10 cc syringe. You see that could be recycled, apart from the catheter. The pump can be re-used.“* – Medical officer

***Recommendations for improving the integration of balloon tamponade into the PPH pathway***

*“I think a lot of it is training. A lot of us are not [formally] trained on it. I don’t think we get to be trained practically. A lot of us have the theory, but the actual practice, the actual practical training is not very common. I would think that even here, a lot of people might not know about it because they are not exposed to it. A lot of it is theory rather than practice.”* – Obstetrician

*“The best way to use UBT, I think, is to have as many people who are delivering mothers – from the midwives to the doctors to the obstetricians– to have a clue or knowledge of UBT, so that when there is an emergency, you can go ahead and try. You can’t know if it works unless you try it. You can’t try it unless you know about it. And the only way you know about it is if you are trained. There are people who have read about it, but who were not trained, and you can’t just try it if you don’t know about the outcome. This is life and death. So I think people need to be aware of it; people need to be trained. People need to know that if a mother is bleeding and I have tried other methods, then I will insert the UBT. But people will never think about it if they have never tried it.”* – Medical officer

*“The pre-assembled {ESM-UBT} kit is easier because it is quicker when you need it. Because usually it is an emergency. Because it takes time to think, where am I going to get this, where am I going to get that? It takes time. But when you have it already, then it is quite easy.”* – Obstetrician

*“I think the best thing is if we can have a complete set so that once you open the kit you don’t have to move looking for this and that. You have everything there. It will be very easy to assist the mother. You see, trying to assemble these things, I lost the 20 minutes that I would have maybe reduced the amount of bleeding. Because the time that I am struggling to find these things, she is still bleeding you see. If there is a complete set, it will be faster in saving lives of these ladies. That would be my wish if the kit comes complete, you don’t have to go look for this go look for this. I think it would be the best.”* – Nurse-midwife
